# CIAPIN1 functions as a redox-sensitive transcriptional repressor of *Tp53* during vascular remodeling

**DOI:** 10.7150/thno.124965

**Published:** 2026-04-23

**Authors:** Seongpyo Lee, Joo-Hui Han

**Affiliations:** 1 College of Pharmacy, Woosuk University, Wanju 55338, Republic of Korea.; 2 College of Pharmacy, Chungbuk National University, Cheongju 28160, Republic of Korea.

**Keywords:** CIAPIN1, p53, reactive oxygen species, intimal hyperplasia, PDGF-BB

## Abstract

**Background:**

Phenotypic switching in vascular smooth muscle cells (VSMCs) is a major driver of pathological vascular remodeling, including atherosclerosis and restenosis. Although p53 is a key regulator of VSMC homeostasis, the precise molecular mechanisms responsible for suppressing p53 expression during growth factor-induced phenotypic transitions remain incompletely understood. The present study identifies cytokine-induced apoptosis inhibitor 1 (CIAPIN1) as a novel redox-sensitive regulator of the p53 signaling axis.

**Methods:**

Primary cultured VSMCs and a rat carotid balloon injury model were used to investigate the role of CIAPIN1 in VSMC dynamics. CIAPIN1 expression was modulated using both gain- and loss-of-function approaches. The interaction between CIAPIN1 and the *Tp53* promoter was examined by dual-luciferase reporter and chromatin immunoprecipitation (ChIP) assays. Redox-dependent subcellular trafficking of CIAPIN1 was assessed using N-acetylcysteine (NAC), Ivermectin (IVM) and site-directed mutagenesis of the nuclear localization signal (NLS).

**Results:**

In human atherosclerotic lesions, CIAPIN1 expression was markedly upregulated, whereas *Tp53* mRNA levels were significantly lower compared with healthy controls. Stimulation with PDGF-BB increased CIAPIN1 expression and the induced CIAPIN1 protein directly bound to specific consensus sites in the *Tp53* promoter, thereby repressing its transcription. This CIAPIN1-mediated suppression of p53 promoted a switch toward the synthetic phenotype in VSMCs, accelerating proliferation and migration. These effects were substantially attenuated by CIAPIN1 knockdown or by restoring p53 expression. Mechanistically, PDGF-BB triggered reactive oxygen species (ROS) production, which promoted the nuclear translocation of CIAPIN1 through a functional C-terminal NLS (residues 236-239) via the classical importin-α/β pathway. Blocking this trafficking axis with the ROS scavenger NAC, the importin inhibitor IVM or genetic deletion of the NLS (∆236-239) restored p53 expression and significantly reduced neointimal formation *in vivo*.

**Conclusions:**

CIAPIN1 functions as a redox-sensitive transcriptional repressor of *Tp53*, driving VSMC phenotypic switching and neointimal hyperplasia. These findings highlight CIAPIN1 as a promising and specific therapeutic target for the treatment of proliferative vascular disorders.

## Introduction

Vascular smooth muscle cells (VSMCs) play as a central role in the development of intimal proliferation, a hallmark of various occlusive vascular diseases such as atherosclerosis, post-angioplasty restenosis, venous graft stenosis and graft arteriopathy 1-3. In healthy arteries, VSMCs in the tunica media maintain a quiescent, contractile phenotype characterized by high expression of contractile proteins, including smooth muscle α-actin (SMA) and calponin. These proteins help regulate vascular tone and preserve vessel wall integrity 4. However, following vascular injury or exposure to mitogenic factors such as platelet-derived growth factor-BB (PDGF-BB), VSMCs undergo a phenotypic switching. They shift toward a synthetic, proliferative state characterized by increased proliferation, migration and extracellular matrix (ECM) remodeling 5-7. This dedifferentiation process drives pathological vascular remodeling, leading to luminal narrowing and the progression of occlusive diseases 8-10. Despite its clinical importance, the molecular mechanisms that control this phenotypic plasticity are still not fully understood, and further studies are needed to identify new regulatory pathways.

Cytokine-induced apoptosis inhibitor 1 (CIAPIN1), also known as anamorsin, is an evolutionarily conserved anti-apoptotic protein localized in the nucleus, cytoplasm, and mitochondria 11. Unlike classical apoptosis regulators such as B-cell lymphoma 2 (Bcl-2) or caspases 12-14, CIAPIN1 is widely expressed across tissues and is upregulated by various growth factors, including PDGF-BB. This upregulation enhances resistance to programmed cell death and influences cellular behavior 15, 16. Recent studies suggest that CIAPIN1 participates in the regulation of VSMC proliferation, migration, and phenotypic modulation 15, 17. Mechanistically, CIAPIN1 activates the Janus kinase 2/signal transducer and activator of transcription 3 (JAK2/STAT3) signaling pathway and represses transcription of the tumor suppressor p53, which is a critical regulator of cell cycle arrest and apoptosis 15. Nevertheless, the precise mechanism by which CIAPIN1 controls VSMC phenotypic switching, particularly in response to vascular injury, has not been completely clarified.

PDGF-BB is a potent mitogen and chemoattractant for VSMCs. It acts by binding to platelet-derived growth factor receptor-β (PDGFR-β), which activates downstream signaling cascades that downregulate contractile markers (SMA, calponin) and promote the synthetic phenotype 18, 19. This phenotypic transition is closely linked to the production of reactive oxygen species (ROS) 20, largely through upregulation of NADPH oxidase 1 (NOX1) 21. NOX1 generates superoxide (O₂⁻) by using NADPH as an electron donor, thereby increasing oxidative stress and accelerating VSMC dedifferentiation and vascular remodeling 22, 23. The resultant oxidative environment strengthens signaling pathways that favor proliferation and migration, worsening vascular pathology. Although the involvement of CIAPIN1 in JAK2/STAT3 activation is well established, its relationship with p53 under ROS-mediated signaling and its specific contribution to VSMC phenotypic modulation remain poorly defined 24. Notably, ROS-induced oxidative stress has been suggested to promote nuclear translocation of CIAPIN1 25, which may in turn influence *Tp53* transcriptional activity and ultimately determine VSMC fate. This possibility warrants detailed investigation.

In the present study, we sought to clarify the molecular mechanisms by which CIAPIN1 regulates *Tp53* transcription during VSMC phenotype switching and vascular remodeling. Using a rat model of neointimal hyperplasia together with primary VSMCs, we examined how PDGF-BB-induced ROS signaling promotes nuclear translocation of CIAPIN1 and subsequent repression of p53. Our results uncover a previously unrecognized PDGF-BB/ROS/CIAPIN1/p53 axis, offering new insights into VSMC plasticity and identifying potential therapeutic targets for proliferative vascular disorders.

## Materials and Methods

### Materials

Fetal bovine serum (FBS), Dulbecco's modified Eagle's medium (DMEM), trypsin-EDTA and antibiotics (penicillin/streptomycin) were purchased from Thermo Fisher Scientific (Gibco, Inc., Waltham, MA, USA). Phosphate-buffered saline (PBS) was obtained from Welgene (Gyeongsan, Korea). Recombinant human PDGF-BB was acquired from PeproTech (Rocky Hill, NJ, USA). Ivermectin was purchased from Sigma-Aldrich (St. Louis, MO, USA). The dual-luciferase reporter assay system was obtained from Promega Corporation (Madison, WI, USA). Detailed information on the antibodies used in this study is listed in Table S1. All other reagents were obtained from Sigma-Aldrich (St. Louis, MO, USA) unless otherwise specified.

### Animals

Male Sprague-Dawley rats (10 weeks old, weighing 380-420 g) were purchased from Samtako (Osan, Korea). Male rats were selected to ensure consistency with prior vascular remodeling studies and to minimize hormonal variability associated with female rats. The rats were housed three per cage under controlled conditions (22 ± 2 °C and 50 ± 5% relative humidity) with a 12/12 h light/dark cycle (lights on at 07:00). A standard rodent chow (1314 FORTIFIED; Altromin, Lage, Germany) and water were provided to the rats on an ad libitum basis. All animals were allowed a 7 days acclimatization period before the experiments to minimize stress. All procedures were approved by the Institutional Animal Care and Use Committee (IACUC) of Woosuk University (Approval No. WS-2023-16), and conducted in accordance with the ARRIVE guidelines 26 and the Guide for the Care and Use of Laboratory Animals 27.

### *In vivo* randomization and blinding procedures

Sample size was determined based on 80% power, α = 0.05 (two-sided), and effect size = 0.8 (the effect size was set relied on pre-experimental results of neointimal area and this type of experiment in our laboratory) by a priori power calculation with G-Power 3.1.9 software (http://www.gpower.hhu.de/). This calculation indicated a minimum sample size of 6 rats per group; we used 7 rats per group to account for potential variability. Randomization was performed using a random number table. All animal experiments were conducted in a blinded manner. Each rat was assigned a temporary random number based on its weight range. After randomization into groups, rats were given permanent numerical designations in their cages. For each group, a cage was selected randomly from the pool of all cages. All outcome assessments were conducted in a blinded manner by two investigators unaware of group assignments or experimental conditions. Data analysis was also performed by investigators blinded to the group allocation.

### Intervention administration

After a 1-week acclimatization period, rats were randomly divided into groups as described in the following section.

### Induction of neointima formation and lentiviral transduction models

A carotid artery balloon injury procedure was conducted in SD rats, following previously established protocols 15, 17. Briefly, rats were anesthetized with pentobarbital sodium (50 mg/kg) via intraperitoneal injection. To perform the mechanical injury, we carefully advanced a 2F Fogarty balloon catheter (Edwards Lifesciences, Irvine, CA, USA) into the left common carotid artery lumen through an incision in the external carotid branch. For the sham group, the carotid artery was surgically exposed without catheter insertion or balloon inflation. To modulate gene expression at the injury site, 50 μL of lentiviral vectors (2×10⁹ PFU/mL) was delivered into the damaged arterial segment. The viral vectors designed to express rat CIAPIN1 (rCIA1), CIAPIN1 shRNA (shCIA1), or control vector (Ctr and Scr) were applied to the injured artery for 30 min. Additionally, 200 μL of rat *Tp53*-specific siRNA (si-*Tp53*, 200 nM) or control siRNA (si-Con), premixed with siPORT NeoFX reagent (Thermo Fisher Scientific), was applied for 30 min post-injury in designated groups. For pharmacological intervention, N-acetylcysteine (NAC, 600 mg/kg) or vehicle (normal saline) was administered orally daily for 14 days, starting 1 day post-BI.

Two cohorts were used (n = 7 per group) to accommodate the different combinations of interventions. Cohort 1: (1) Sham+Scr+si-Con, (2) BI+Scr+si-Con, (3) BI+shCIA1+si-Con, (4) BI+shCIA1+si-*Tp53*. Cohort 2: (1) Sham+Ctr, (2) Sham+rCIA1 (3) BI+Ctr, (4) BI+rCIA1, (5) BI+rCIA1+NAC. Inclusion criteria required healthy male rats (10 weeks, 380-420 g) with no signs of illness post-acclimatization. Exclusion criteria included surgical complications (excessive bleeding, catheter failure) or post-operative infections. During surgery, respiratory rate and body temperature were monitored. Post-operative management consisted of wound closure with skin sutures, disinfection with povidone-iodine, recovery on a thermal mat and administration of 1% ketoprofen (0.2 mL/kg) as an analgesic with daily wound inspection. Rats were monitored daily for distress, weight loss, or infection. At 14 days post-BI, rats were euthanized by CO_2_ overdose. Carotid arteries were harvested, snap-frozen in liquid nitrogen for gene/protein expression analyses, or fixed in 4% paraformaldehyde for histological staining.

### Microarray analysis

Gene expression data were obtained from human GEO database (GSE40231; coronary and carotid artery disease) 28, which compared the gene expression profiles of left and right from coronary arteries bypass grafting (CABG) patients to identify differentially expressed gene associated with atherosclerosis. RNA samples from coronary arteries were obtained from 40 patients, along with samples from atherosclerotic arterial walls and non-atherosclerotic arterial wall. In the validation cohort, 25 carotid lesions from 39 patients were randomly selected for RNA isolation and gene expression profiling.

### Haematoxylin and eosin (H&E)

Arterial cross-sections (4 μm thick) were prepared from paraffin-embedded tissues and stained with hematoxylin and eosin (H&E) 29. Images were captured using a ZEISS Axio Vert.A1 TL/RL-LED microscope (ZEISS Instrument Inc., Jena, Germany). Histological evaluations were performed using ImageJ software (Version 1.54g) to measure residual lumen area, areas enclosed by the internal and external elastic lamina, intimal area, and intima-to-media area ratio to quantify neointimal thickening.

### Cell culture

Rat primary aortic VSMCs were obtained from Cell Applications, Inc. (Cat# R354-05a), while HEK293T cells were obtained from ATCC (Cat# CRL11268, RRID:CVCL_1926) as previously described 30. Both cell types were maintained in DMEM enriched with 10% (v/v) FBS, 100 IU/mL penicillin, and 100 μg/mL streptomycin. The cultures were kept at 37 °C within a humidified environment composed of 95% air and 5% CO_2_. Cells within passages 4-7 were utilized for experiments. Human primary aortic smooth muscle cells (HASMCs, PCS-100-012™) were obtained from ATCC (Manassas, VA, USA). HASMCs were cultured in complete media (vascular cell basal medium [ATCC PCS-100-030™]) supplemented with vascular smooth muscle cell growth kit (ATCC PCS-100-042™), 100 IU/mL penicillin, and 100 μg/mL streptomycin at 37 °C in a moisture-saturated incubator containing 5% CO_2_. For all experimental sets, we utilized between passages 4-6.

### Plasmid construction

The pLVX-EF1α-IRES-Puro lentiviral expression system was sourced from Takara Bio Inc. (CA, USA). The rat *Ciapin1* was acquired from Origene Technologies (Rockville, MD, USA). Konrad Buessow (Addgene, RRID: Addgene_34811) generously provided the pQTEV-LOC57019 plasmid, which harbors the 39 kDa human *CIAPIN1* (hCIAPIN1) insert. The full-length human *CIAPIN1* coding sequence was subsequently sub-cloned into the destination lentiviral vector. To investigate the functional role of the nuclear localization signal (NLS), an NLS-deleted mutant of human CIAPIN1 (△236-239) was generated. This mutant was constructed using the pQTEV-LOC57019 plasmid as a template and primers (Table S5). Rat *Ciapin1* shRNA lentiviral plasmids, including scrambled sequences, were obtained from Origene Technologies. A series of pGL2-based luciferase reporter constructs containing the *Tp53* promoter (-344 to +12, -188 to +12 and -38 to +1) relative to the primary transcription start site were gift from Wafik El-Deiry (Addgene, RRID:Addgene_16292).

### Lentivirus production

The lentiviral packaging plasmids psPAX2 (Addgene, plasmid Cat# 12260, RRID:Addgene_12260) and pMD2.G (Addgene, plasmid Cat# 12259, RRID:Addgene_12259) were co-transfected with the genomic plasmid into HEK293T cells (Cat# CRL11268, RRID:CVCL_1926) to generate recombinant viral particles 31. Lenti-X p24 Rapid Titer Kit (Takara Bio Inc.) used to quantify the virus titers. Subsequently, VSMCs were transduced with rat *Ciapin1* shRNAs (sh*CIA1* #1 and #3), along with appropriate controls.

### Western blot analysis

To prepare protein extracts, cells were disrupted on ice using a RIPA lysis buffer comprising 50 mM Tris-HCl (pH 8.0), 150 mM NaCl, 1.0% NP-40, 2 mM EDTA, 5 mM NaF, 1 mM PMSF, 1 mM Na_3_VO_4_, 0.5% sodium deoxycholate, and 0.1% SDS. The protein content within the resulting cleared supernatants was quantified via a BCA protein assay kit (Pierce, Rockford, IL, USA). Equal protein masses were resolved via SDS-PAGE and subsequently electroblotted onto PVDF membranes (ATTO Corp., Tokyo, Japan). Following a 1 h blocking period in TBS-T (10 mM Tris, 150 mM NaCl, 0.1% Tween-20, pH 7.6) containing 5% BSA, the membranes were probed with primary antibodies overnight at 4 °C. This was followed by secondary antibody incubation (Table S1), with antibodies diluted in the same blocking buffer for 6 h at 4 °C. Immunoreactive bands were visualized through an enhanced chem-iluminescence system (ECL, ATTO Corp., Tokyo, Japan). β-actin used as a control for normalization, and we performed densitometric quantification of the bands by Image Lab software (Version 6.1, Bio-Rad, Hercules, CA, USA).

### Immunofluorescence staining analysis

Immunofluorescence staining was performed as described previously 32. Dual immunofluorescence staining was performed on rat carotid artery sections and VSMCs using CIAPIN1 and p53 antibodies. Arterial sections were imaged using a confocal laser microscope (Carl Zeiss, LSM 980, Germany). VSMCs were seeded on coverslips in 24-well plates, fixed with formaldehyde, permeabilized with 0.25% Triton X-100 in PBS with 1% BSA for 5 min, and blocked with 5% goat serum in PBS for 1 h. Cells were incubated with primary antibodies for 15 h at 4 °C, followed by secondary antibodies in PBS with 3% BSA for 2.5 h at room temperature. Nuclei were stained with DAPI, and images were captured using a confocal microscope (Carl Zeiss, LSM 980). Z-stack images were created by merging serial 4 μm scans.

### Small-interfering RNAs transfection

We employed an established methodology for small-interfering RNAs transfection 33. The rat-specific siRNA duplexes targeting *Tp53* (si-*Tp53*; GenBank accession no. NM_030989.3) and the non-targeting scrambled control (si-Con) were synthesized by Bioneer Inc. (Daejeon, Korea). For transfection, VSMCs were incubated with 30 nM siRNA in Opti-MEM (Thermo Fisher Scientific, Waltham, MA, USA) with Lipofectamine 2000 (Invitrogen) for a 4 h period. Knockdown efficiency was verified by immunoblot analysis of whole-cell lysates. The specific nucleotide sequences for the siRNAs are detailed in Table S2.

### Cell proliferation and viability assays

The proliferation of VSMCs was assessed via a colorimetric determination using the 3-(4,5-dimethylthiazol-2-yl)-2,5-diphenyltetrazolium bromide (MTT) assay (AMRESCO LLC, OH, USA). Following a 24 h period of serum deprivation to achieve cell cycle synchronization, cells with modulated CIAPIN1 expression (overexpression or knockdown) were stimulated with PDGF-BB (30 ng/mL). Where indicated, cells were also pre-treated with pifithrin-α (10 μM) or si-*Tp53*. After 24 h treatment period, 5 mg/mL of MTT solution was introduced to the culture for 3 h incubation at 37 °C. To quantify the metabolic activity, the generated formazan crystals were solubilized in dimethyl sulfoxide, and the optical density was measured at 565 nm using a microplate reader (Tecan Group Ltd., Männedorf, Switzerland). All data are presented as fold changes relative to the untreated control group (without PDGF-BB stimulation).

### Boyden chamber assay

VSMCs migration was evaluated using Boyden chamber assays. For this assay, 48-well reusable chambers (Neuroprobe, Gaithersburg, MD, USA) with polyvinyl pyrrolidone-free (PVPF) membranes (8.0 μm; GE Water & Process Technologies, Trevose, PA, USA) coated with type I collagen (VWR International, Cat# 354236, West Chester, PA, USA) were employed. Serum-starved VSMCs with CIAPIN1 overexpression or knockdown in 60 mm dishes were trypsinized and suspended in serum-free media. Cells were then added to the upper chamber at a density of 5×10^4^ cells/well and allowed to migrate towards the PVPF membranes and the lower chamber containing medium with or without PDGF-BB (30 ng/mL) for 24 h at 37 °C. Subsequently, membranes were stained using the Diff-Quik solution kit (Sysmex, Cat# 38721, Kobe, Japan) following the manufacturer's instructions. The stained membranes were air-dried on a microscope slide, and non-migrated cells were removed by wiping the membranes with cotton swabs. Images were captured using a Zeiss microscope (Axio vert A1, Zeiss, Germany), and the number of migrated cells was counted and averaged across five randomly selected fields per membrane.

### Quantitative real-time PCR (qPCR)

For the analysis of RNA level, we used qPCR as described previously 31, 34. Total RNA was isolated from both cellular and tissues samples utilizing the TRIzol™ (Invitrogen, CA, USA) according to the manufacturer's instructions. To synthesize cDNA, 1 μg of isolated RNA was reverse-transcribed within a thermocycler using the AccuPower RT Premix (Bioneer, Daejeon, Korea). Quantitative amplification was then carried out on 96-well optical plates, utilizing the SYBR Green qPCR Premix (Toyobo, Osaka, Japan) in a final reaction volume of 20 μL, which included 400 nM of each primer. Fluorescence signals were captured using a CFX96 Real-Time Detection System (Bio-Rad, Hercules, CA, USA). The thermal profile was programmed with an initial 3 min denaturation at 95 °C, followed by 39 cycles of 95 °C for 10 s and 60 °C for 30 s. For the melt-curve analysis, the temperature was increased from 65 °C to 95 °C in 0.5 °C increments every 5 s. To ensure the accuracy of our data, we only analyzed results from primer pairs that generated a singular amplicon of the predicted molecular weight. Primer sets used in this study are listed in Table S3. Relative gene expression was calculated using the 2^-∆∆ct^ method, 35 normalized to β-actin.

### Dual-luciferase reporter assay

VSMCs were co-transfected with a genetic mixture containing 1.25 μg of the pGL2-*p53* promoter reporter (firefly luciferase) and 7.5 ng of the pRL-TK* Renilla luciferase* internal control (Thermo Fisher Scientific, Waltham, MA, USA) utilizing Lipofectamine 2000. Following a 4 h incubation period post-transfection, the cells underwent transduction with either human CIAPIN1 or rat-specific shRNA targeting *Ciapin1* (shCIA1 #1). After 48 h, cell cycle synchronization was achieved by maintaining the cells in serum-free medium for 24 h, followed by stimulation with 30 ng/mL PDGF-BB for the specified durations. Post-treatment, cellular lysates were prepared using passive lysis buffer, and the bioluminescent signals were analyzed through the Dual Luciferase Reporter Assay System (Promega, Madison, WI, USA) using a Glomax 20/20 luminometer. The *Tp53* promoter activity was determined by normalizing the firefly luciferase signals against the *Renilla luciferase* internal control. Under identical experimental conditions excluding the serum starvation step, *Tp53* promoter activity was also evaluated in CIAPIN1-overexpressing HASMCs.

### Chip-PCR assay

CIAPIN1 overexpressing VSMCs were treated with NAC (5 mM, positive control for antioxidant activity) for 30 min and stimulated with H_2_O_2_ (100 μM) for 1 h or PDGF-BB (30 ng/mL) for 3 or 8 h. The SimpleChIP® Enzymatic Chromatin IP Kit (Cell Signaling Technology) was used for the ChIP assay, according to the manufacturer's guidelines, followed by PCR. The primers used for PCR are listed in Table S4.

### Reactive oxygen species (ROS) generation assay

To evaluate intracellular ROS levels, we used a fluorescent probe, H_2_DCFDA, as previous described 31. VSMCs were treated with or without NAC (5 mM) for 30 min before incubation with H_2_O_2_ (100 μM, 1 h) or PDGF-BB (30 ng/mL, 3 h) in black 96-well plates. Cells. Once the specific treatment period ended, the culture media were discarded. The VSMCs were then loaded with 20 μM H_2_DCFDA, which had been prepared in serum-free medium, for a 30 min incubation period at 37 °C. Upon removal of the unbound probe, the cells were washed and maintained in PBS, and the fluorescence intensity (Ex/Em = 485/530 nm) was quantified using an Infinite F200 microplate reader (Tecan Group Ltd., Männedorf, Switzerland). For comparative analysis, the resulting values were normalized and expressed as fold changes relative to the unstimulated control group. To visualize superoxide production *in situ*, carotid artery cross-sections were incubated with 10 μM dihydroethidium (DHE; Invitrogen, Carlsbad, CA, USA). Arteries were immediately embedded in OCT, sectioned at 4 μm, and mounted onto glass slides. Slides were covered with 10 μM of DHE solution and incubated for 30 min in a light protected humidified chamber at 37 °C. Then, the sections were coverslipped and observed under a ZEISS Axio Vert.A1 TL/RL-LED microscope (ZEISS Instrument Inc., Jena, Germany). The quantification of DHE-positive cells was performed by evaluating no fewer than five stochastic microscopic fields per sample across three independent biological trials, and fluorescence intensity was subsequently processed using ImageJ software (Version 1.54g).

### Measurement of glutathione (GSH) levels

Cell monolayers were harvested in PBS and centrifuged at 1,000 g at 4 °C for 5 min. The harvested cell pellets were lysed in a specialized extraction buffer (0.1% Triton-X, 0.6% sulfosalicyclic acid in 0.1 M potassium phosphate buffer with 5 mM ethylenediaminetetracetic acid, pH 7.5). The lysates were subjected to 3 min sonication with vertexing every 30 s, followed by centrifugation at 3,000 g for 5 min at 4 °C. To quantify total GSH, the resulting supernatants were reacted with a mixture containing 5,5-dithio-bis (2-nitrobenzoic acid) (0.66 mg/mL), glutathione reductase (3 U/mL) and NADPH (0.66 mg/mL). Absorbance was recorded at 412 nm using a microplate reader. GSH concentrations were determined against a standard curve and corrected for the total protein content of each sample, as measured by the Bradford assay.

### Cell fractionation

Cellular fractions were isolated following a previously described method 17. To obtain distinct cellular components, VSMCs were harvested and spun at 800 g for 10 min after the reaction was terminated. The resulting cell pellets were thoroughly homogenized using an ice-cold lysis buffer (Buffer 1: 50 mM Tris-HCl, 250 mM sucrose, pH 7.4, 5 mM MgCl_2_, 2 mM EDTA, 5 mM NaF, 1 mM PMSF, and 1 mM Na_3_VO_4_). Following a 30 min incubation on ice and 15 s of vortexing, the mixture was centrifuged at 800 g for 15 min at 4 °C to separate the cytoplasmic and nuclear fractions. The pellet was subjected to an additional wash in Buffer 1 and spun at 500 g for 15 min at 4 °C. For nuclear protein extraction, the pellet was resuspended in a high-salt buffer (Buffer 2: pH 7.9, 20 mM HEPES, 1.5 mM MgCl_2_, 0.5 M NaCl, 20% glycerol, 1% Triton X-100, 2 mM EDTA, 5 mM NaF, 1 mM PMSF and 1 mM sodium orthovanadate) After a 30 min ice incubation, the samples were disrupted via Vibra-Cell sonicator (Sonics, Newtown, CT, USA) using 5 s pulses with 10 s intervals. Finally, the nuclear supernatant was collected after a 9,000 g spin for 30 min at 4 °C and prepared for western blot analysis with 5×SDS loading buffer. Lamin A/C was utilized to confirm the purity and loading of the nuclear extracts.

### Statistical analyses

Data were expressed as mean ± standard error of the mean (S.E.M.) from at least three independent experiments. Statistical analyses were conducted using GraphPad Prism Software (Version 10, San Diego, CA, USA). Differences between two groups were analyzed using a two-tailed unpaired Student's t-test. Comparisons among three or more groups were performed using one-way analysis of variance (ANOVA), followed by Bonferroni's post hoc test when the overall ANOVA was statistically significant (P < 0.05) and the assumption of homogeneity of variance was satisfied, as assessed by Bartlett's test. Correlations were analyzed using Pearson's correlation test. P-value threshold of less than 0.05 was defined as the requirement for statistical significance. The levels of significance are represented using the following notation: ^*^P < 0.05, ^**^P < 0.01, ^***^P < 0.001, and ^****^P < 0.0001.

## Results

### p53 suppression counteracts the CIAPIN1 knockdown-induced attenuation of neointima formation

**Figure 1:**
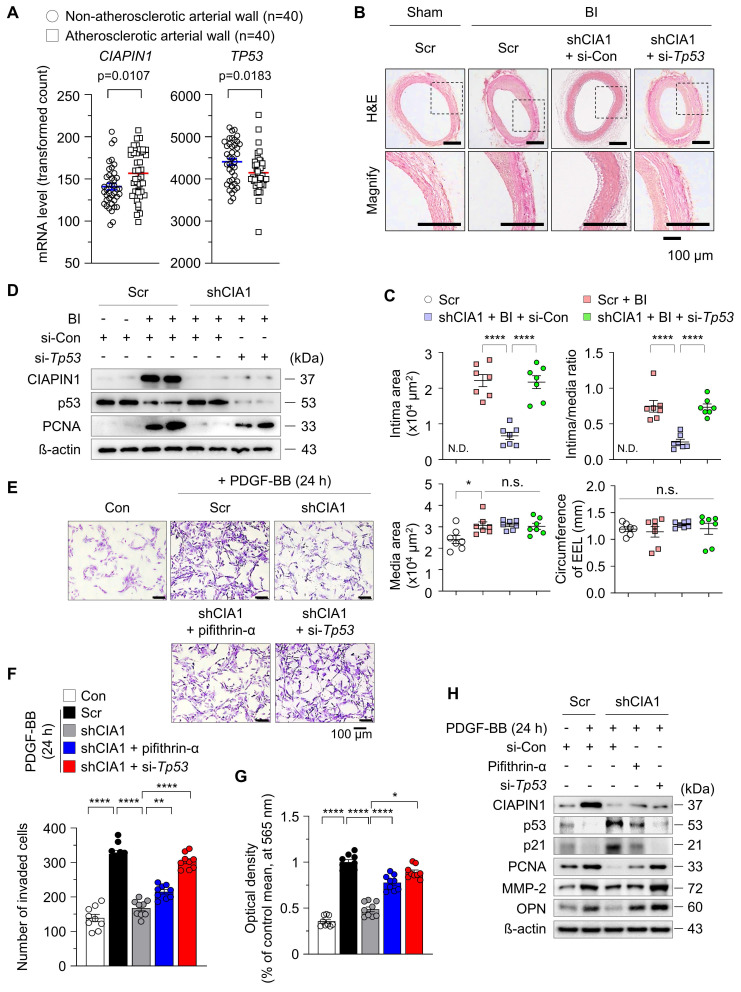
** CIAPIN1 regulates vascular smooth muscle cell (VSMC) phenotypic switching and neointimal formation via p53 suppression.** (**A**) Expression levels of* CIAPIN1* and *Tp53* were analyzed in human carotid artery specimens with or without atherosclerosis (n = 40 per group). **(B)** Representative hematoxylin and eosin (H&E) staining of carotid arteries from a balloon injury (BI) rat model, showing neointimal formation in scramble control (Scr), shCIAPIN1 (shCIA1), shCIAPIN1 + si-Con (shCIA1 + si-Con), and shCIAPIN1 + si-*Tp53* (shCIA1 + si-*Tp53*) groups. Scale bar: 100 μm. I: intima, M: media. **(C)** Quantification of intima area (left) and intima-to-media ratio (right) in carotid arteries from the groups in (B) data from (C) are presented as mean ± SEM (n = 7). **(D)** Western blot analysis of CIAPIN1, p53, and PCNA in carotid arteries with BI, Scr, shCIAPIN1, si-Con, and si-*Tp53*. β-actin was used as a loading control. Cell migration and proliferation were assessed using **(E-F)** transwell assays and **(G)** MTT assays, and in CIAPIN knockdown VSMCs following stimulation with pifithrin-α (p53 inhibitor), si-Con, si-*Tp53*, and PDGF-BB (30 ng/mL) for 24 h. Scale bar: 100 μm. **(H)** Western blot analysis of CIAPIN1, p53, p21, PCNA, MMP-2, and OPN in CIAPIN knockdown VSMCs after treatment with pifithrin-α, si-Con, si-*Tp53*, and PDGF-BB (30 ng/mL) stimulation for 24 h. ^*^p < 0.05, ^**^p < 0.01, ^****^p < 0.0001 *vs*. each group. n.s.: not significant.

To assess the relevance of CIAPIN1 and p53 in vascular biology and pathophysiology, we analyzed the human GEO database (GSE40231; coronary and carotid artery disease), which is publicly available. Our analysis revealed a significant elevation in *CIAPIN1* mRNA levels in the atherosclerotic arterial walls (AAWs) compared to levels in the non-atherosclerotic arterial walls (NAAWs) of patients, however *TP53* mRNA levels were reversed like previous *in vitro* results (Figure 1A). To validate these clinical observations and investigate the functional impact, we induced balloon injury (BI) in the rat common carotid artery and administered either *Ciapin1*-targeting shRNA (shCIA1) with scramble control (Scr) or *Tp53*-specific siRNA (si-*Tp53*) with siRNA control (si-Con). BI-induced neointima formation was significantly attenuated by CIAPIN1 inhibition. However, concurrent suppression of *Tp53* reversed this inhibitory effect, suggesting that CIAPIN1 and *Tp53* exert opposing influences on neointima formation (Figure 1B-C). Our findings indicate that the upregulation of CIAPIN1 following balloon injury serves to repress p53 expression while concurrently increasing proliferating cell nuclear antigen (PCNA) levels. Conversely, pharmacological or genetic inhibition of CIAPIN1 successfully restored p53 expression and reduced PCNA levels (Figure 1D, Figure S1). Inhibition of p53 counteracted these effects, reinstating the pro-proliferative changes induced by BI. At the cellular level, CIAPIN1 suppression significantly inhibited platelet-derived growth factor-BB (PDGF-BB)-induced vascular smooth muscle cell (VSMC) proliferation and migration, and these effects were reversed by *Tp53* knockdown using si-*Tp53* or by pharmacological inhibition using pifithrin-α (Figure 1E-G). Moreover, CIAPIN1 inhibition prevented PDGF-BB-induced reductions in p53 and p21, as well as the associated increases in a proliferation marker PCNA, a migration marker matrix metalloproteinase-2 (MMP-2), and a synthetic phenotype marker osteopontin (OPN) (Figure 1H, Figure S2A-B). These regulatory effects were reversed upon p53 suppression. In summary, our data collectively suggest that CIAPIN1 promotes neointima formation by downregulating p53 levels in VSMCs following vascular injury.

### CIAPIN1 acts as a transcriptional repressor of *Tp53* to regulate the dynamics of VSMC

**Figure 2:**
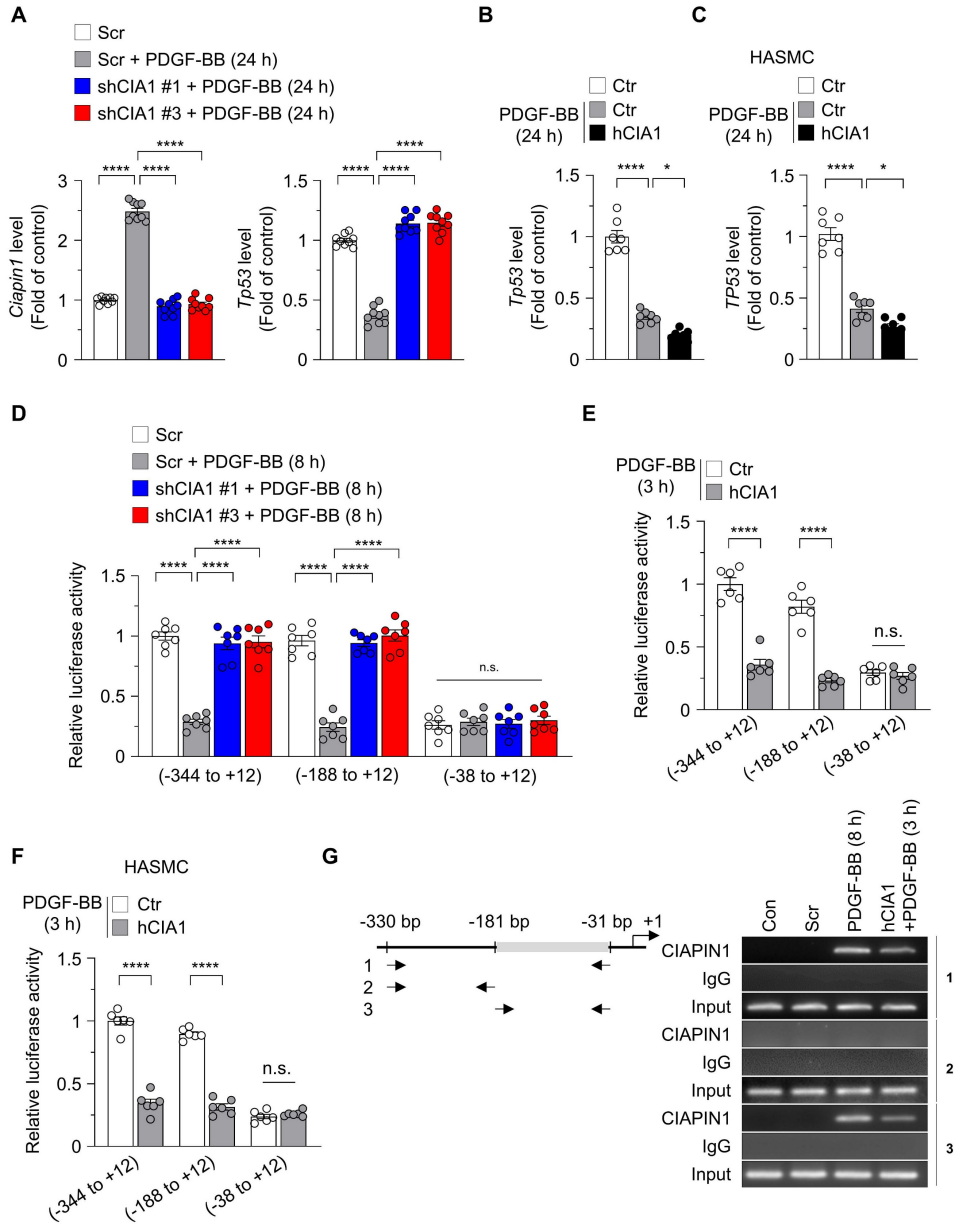
** CIAPIN1 represses *Tp53* transcription by suppressing promoter activity. (A-B)** Real-time analysis showing the mRNA levels of *Ciapin1* and *Tp53* in VSMCs exhibiting CIAPIN1 knockdown **(A)** or overexpression **(B)** after PDGF-BB (30 ng/mL) stimulation for 24 h (n = 9 per group). **(C)** Real-time PCR analysis of *TP53* in HASMCs. **(D-E)** Luciferase reporter assays showing the activities of human *TP53* promoter constructs (pGL2-356, -200, and -50 bp) in VSMCs with CIAPIN1 knockdown **(D)** or overexpression **(E)** after PDGF-BB (30 ng/mL) stimulation for 8 h or 3 h, as indicated. **(F)** Activities of the human *TP53* promoter constructs (pGL2-356, -200, and -50 bp) in HASMCs. Data are presented as fold induction relative to control cells. Firefly luciferase activity driven by the *TP53* promoter reporter was normalized to *Renilla luciferase* activity (n = 6-8 per group).** (G)** Chromatin immunoprecipitation (ChIP)-PCR analysis of CIAPIN1 binding to the *Tp53* promoter in VSMCs overexpressing CIAPIN1 after PDGF-BB (30 ng/mL) stimulation for 8 h. Chromatin immunoprecipitation was performed using an anti-CIAPIN1 antibody or normal rabbit IgG. Input represents chromatin fragments prior to immunoprecipitation. Data represent mean ± S.E.M. values of four independent experiments. ^*^p < 0.05, ^****^p < 0.0001 *vs*. each group. n.s.: not significant.

Transcriptional regulation is a key mechanism in controlling protein expression and plays an important role in determining gene activity and maintaining cellular homeostasis. To investigate the role of CIAPIN1 in modulating *Tp53* transcription during VSMC remodeling, we assessed *Tp53* mRNA expression in VSMCs with altered CIAPIN1 levels under PDGF-BB stimulation. CIAPIN1 knockdown using shCIAPIN1 #1 or #3 significantly upregulated *Tp53* mRNA levels in VSMCs treated with PDGF-BB for 24 h compared to the Scr, identifying CIAPIN1 as a negative regulator of *Tp53* transcription (Figure 2A). In addition, overexpression of human CIAPIN1 (hCIAPIN1) in VSMCs and HASMCs significantly reduced *Tp53* mRNA levels for 24 h after PDGF-BB treatment, supporting the effect of CIAPIN1 in suppressing *Tp53* expression (Figure 2B-C). Consistent with these mRNA expression patterns, overexpression of CIAPIN1 in VSMCs and HEK293T suppressed protein levels of p53 and its downstream effector p21 (Figure S3A-B). Immunofluorescence analysis revealed that CIAPIN1, upregulated by PDGF-BB stimulation, was primarily localized in the nucleus, whereas p53, which increased upon CIAPIN1 knockdown, was primarily present in the cytoplasm (Figure S4). This suggest that CIAPIN1 as a nuclear transcriptional repressor. To explore the mechanistic basis of CIAPIN1-mediated *Tp53* suppression, we performed a dual-luciferase reporter assay to evaluate *Tp53* promoter activity. VSMCs were transfected with luciferase constructs containing *Tp53* promoter fragments spanning -344 to +12, -188 to +12, or -38 to +12 bp and treated with PDGF-BB for 8 h. CIAPIN1 knockdown significantly enhanced *Tp53* promoter activity in constructs harboring the -344 to +12 and -188 to +12 fragments, but this effect was abolished in the -38 to +12 fragment (Figure 2D), indicating that the -188 to -38 bp region is critical for CIAPIN1-dependent repression. Correspondingly, CIAPIN1 overexpression markedly reduced *Tp53* promoter activity in the -344 to +12 and -188 to +12 fragments, but not in the -38 to +12 fragment, following 3 h of PDGF-BB stimulation (Figure 2E). This pattern was recapitulated in HASMCs, where hCIAPIN1 overexpression similarly suppressed *Tp53* promoter activity in the -344 to +12 and -188 to +12 fragments (Figure 2F). Collectively, these results suggest that CIAPIN1 regulates *Tp53* transcription through its influence on the -181 to -31 bp region of the *Tp53* promoter, thereby repressing *Tp53* expression in VSMCs and promoting VSMC dynamics in response to PDGF-BB stimulation. Thus, deletion of the *Tp53* promoter region spanning -181 to -31 markedly reduced CIAPIN1 binding to the *Tp53* promoter, as determined by ChIP assay (Figure 2G). Taken together, these results demonstrate that CIAPIN1 directly represses *Tp53* transcription by binding to a critical proximal promoter region, thereby downregulating p53 signaling and facilitating PDGF-BB-induced VSMC remodeling.

### PDGF-BB-induced ROS generation promotes CIAPIN1 nuclear translocation in VSMCs via an importinα/β- dependent pathway

**Figure 3:**
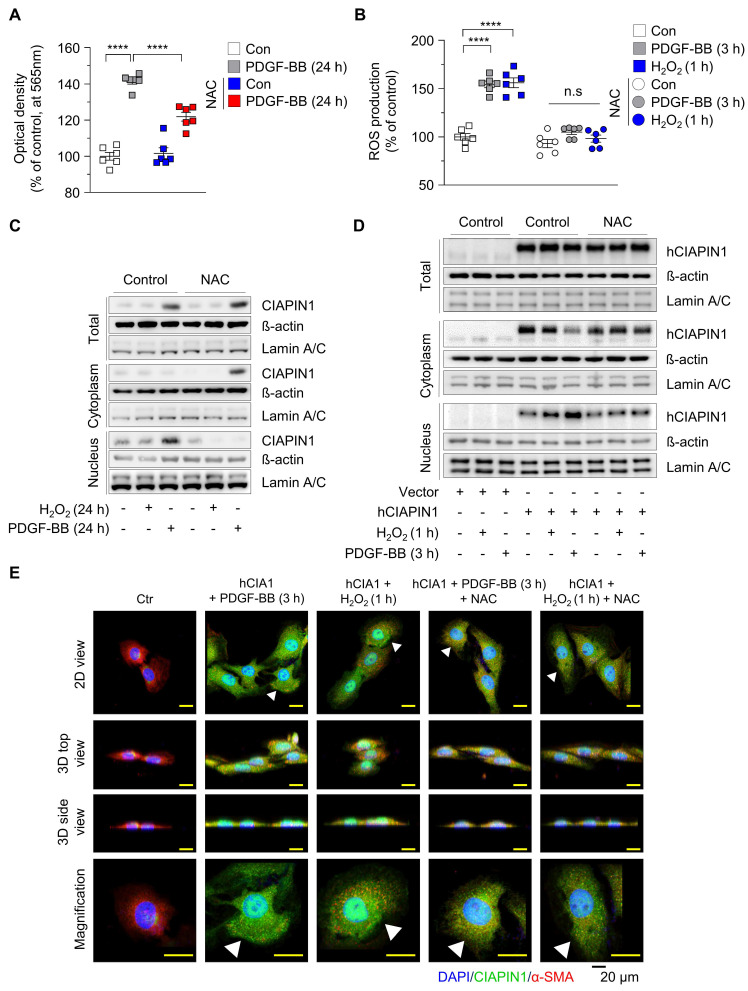
** ROS promotes translocation of CIAPIN1 from the cytosol to the nucleus. (A)** MTT assay of VSMCs pretreated with NAC (5 mM) for 30 min prior to stimulation with PDGF-BB (30 ng/mL) for 24 h. **(B)** Intracellular ROS levels in VSMCs treated with PDGF-BB (30 ng/mL, 3 h) or H₂O₂ (100 μM, 1 h) following pretreatment with NAC (5 mM) for 30 min. ROS production was measured using H_2_DCFDA (n = 6 per group). **(C)** Western blot analysis of CIAPIN1 in cytosolic and nuclear fractions of VSMCs pretreated with NAC (5 mM) for 30 min and then stimulated with H₂O₂ (100 μM) or PDGF-BB (30 ng/mL) for the indicated times. β-actin and Lamin A/C were used as cytoplasmic and nuclear markers, respectively. **(D)** Western blot analysis for CIAPIN1 in subcellular compartments of CIAPIN1 overexpressing VSMCs pretreated with NAC (5 mM) for 30 min, followed by stimulation with H_2_O_2_ (100 μM, 1 h) and PDGF-BB (30 ng/mL, 3 h). **(E)** Representative Z-stack immunofluorescence images showing the localization of CIAPIN1 (green) and α-SMA (red) in CIAPIN1 overexpressing VSMCs pretreated with NAC (5 mM) for 30 min, followed by stimulation with H_2_O_2_ (100 μM, 1 h) or PDGF-BB (30 ng/mL, 3 h). Nuclei were stained with DAPI (blue). Images are presented as 2D view, 3D top view, 3D side view, and magnified view. Scale bars: 20 μm. ^****^p < 0.0001 *vs*. each group. n.s.: not significant.

Vascular injury triggers the release of PDGF-BB, which binds to PDGFR-β on the cell membrane, activating NADPH oxidase and reducing glutathione (GSH) levels, thereby inducing substantial reactive oxygen species (ROS) generation 36. Previous studies have shown that CIAPIN1 undergoes active nuclear translocation in response to ROS, as observed with 6-hydroxydopamine treatment in dopaminergic neuronal cells (MN9D) and rat brain 37. In this study, PDGF-BB stimulation enhanced CIAPIN1-mediated suppression of the p53 axis compared to untreated controls, suggesting that PDGF-BB modulates CIAPIN1 function (Figure S3A). We hypothesized that PDGF-BB-induced ROS generation in VSMCs promotes CIAPIN1 nuclear translocation, thereby influencing *Tp53* transcription and VSMC dynamics. To verify this, we first evaluated whether CIAPIN1 directly affects GSH level to regulate ROS levels. While hCIAPIN1 transfection alone did not alter GSH levels, NAC, a GSH precursor, treatment significantly increased GSH concentrations, indicating that CIAPIN1 by itself is not sufficient to regulate ROS levels (Figure S5). Subsequently, we investigated the effects of PDGF-BB on VSMC proliferation and ROS production. PDGF-BB treatment for 24 h significantly enhanced VSMC proliferation compared to untreated controls, an effect that was markedly attenuated by NAC pretreatment (Figure 3A). To quantify intracellular ROS levels, we performed an H_2_DCFDA assay in VSMCs treated with PDGF-BB (3 h), H₂O₂ (1 h), or NAC. PDGF-BB treatment significantly elevated ROS levels to a degree comparable to H₂O₂ treatment, while NAC pretreatment effectively neutralized this increase (Figure 3B), confirming that PDGF-BB induces robust ROS generation in VSMCs. Given that ROS is established as a driver of CIAPIN1 nuclear translocation, we examined whether PDGF-BB-induced ROS exerts a similar effect. Nuclear and cytosolic fractions were isolated from VSMCs under various conditions: untreated (control), NAC-treated, H₂O₂-treated (24 h), or PDGF-BB-treated (24 h) groups. Western blot analysis revealed that H₂O₂ alone did not significantly alter CIAPIN1 expression in either fraction (Figure 3C). In contrast, PDGF-BB treatment markedly increased total CIAPIN1 expression and increased CIAPIN1 predominant localized to the nuclear fraction (Figure 3C, Figure S6A). Notably, pretreatment with NAC led to the significant retention of CIAPIN1 in the cytoplasm, thereby reducing its nuclear accumulation (Figure 3C, Figure S6A). These findings indicate that ROS generation is a requisite step for CIAPIN1 nuclear translocation in VSMCs. To eliminate potential confounding effects of altered CIAPIN1 expression levels, we overexpressed CIAPIN1 using hCIAPIN1 and assessed its subcellular localization under ROS-inducing conditions. Western blot analysis of nuclear and cytoplasmic fractions showed that H₂O₂ treatment (1 h) induced a modest increase in nuclear CIAPIN1, whereas PDGF-BB treatment (3 h) more robustly drove CIAPIN1 nuclear translocation (Figure 3D, Figure S6B). Pretreatment with NAC effectively blocked this ROS-mediated nuclear influx in both H₂O₂- and PDGF-BB-treated cells (Figure 3D, Figure S6B). Immunofluorescence microscopy further supported these findings, revealing enhanced nuclear localization of CIAPIN1 in hCIAPIN1-overexpressing VSMCs treated with PDGF-BB or H₂O₂. This effect was markedly attenuated by co-treatment with the ROS scavenger NAC (Figure 3E). Cells were co-stained with DAPI for nuclei and α-SMA for cytosol, with 2D, 3D, and magnified views highlighting CIAPIN1's nuclear presence (Figure 3E). To further elucidate the molecular machinery governing CIAPIN1 nuclear trafficking, we investigated the requirement for a specific nuclear localization signal (NLS) and the involvement of the importin-α/β pathway (Figure S7). Sequence analysis identified a putative basic-rich NLS motif (236-KKRK-239; K: Lysine; R: Arginine) within the C-terminal domain (Figure S7A). Genetic deletion of this specific motif (∆236-239) and pharmacological inhibition of importin-α/β-mediated pathway using ivermectin significantly inhibited PDGF-BB-induced nuclear accumulation of CIAPIN1, as confirmed by immunofluorescence microscopy (Figure S7B). Collectively, these findings demonstrate that PDGF-BB-induced ROS facilitates CIAPIN1 nuclear import and that both its C-terminal NLS and the importin-α/β transport machinery are required for CIAPIN1 translocation, thereby enabling *Tp53* transcriptional repression and facilitating VSMC activation during vascular remodeling.

### PDGF-BB-induced ROS generation enhances CIAPIN1-mediated suppression of the *Tp53* promoter and promotes neointima formation

**Figure 4:**
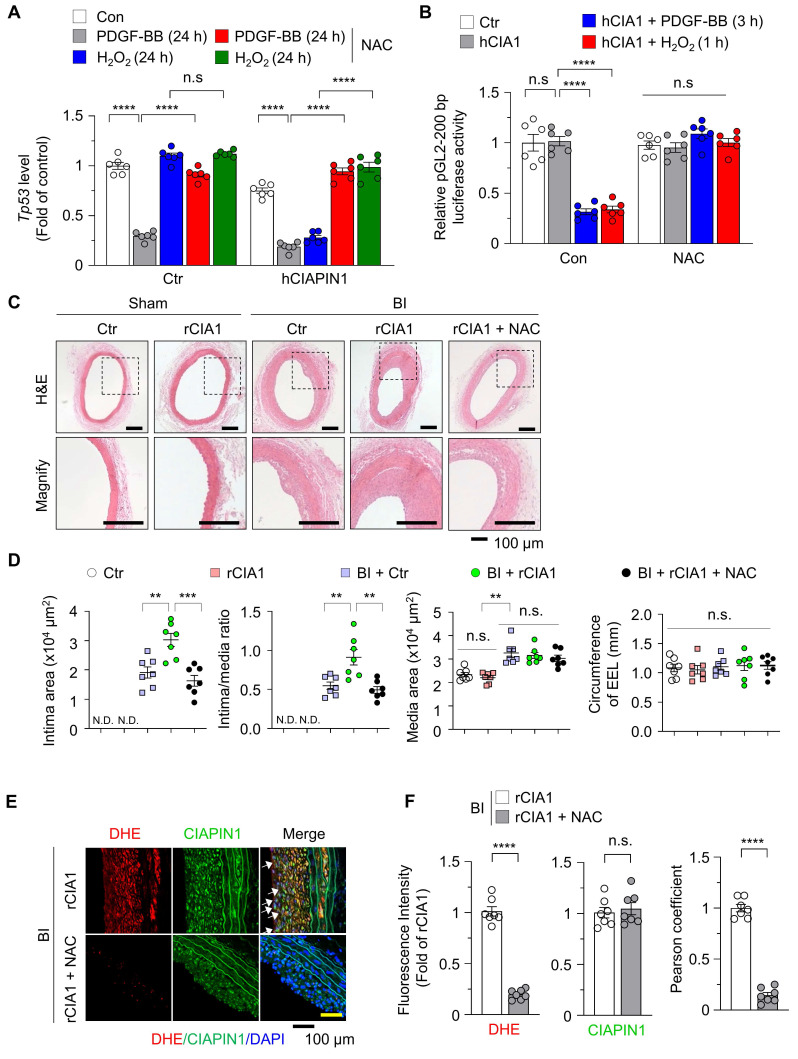
** ROS scavenging inhibits CIAPIN1-mediated vascular hyperplasia. (A)** Real-time PCR analysis of *Tp53* mRNA levels in CIAPIN1-overexpressing VSMCs pretreated with NAC (5 mM) for 30 min and then stimulated with H₂O₂ (100 μM, 24 h) or PDGF-BB (30 ng/mL, 24 h) (n = 6 per group).** (B)** Luciferase reporter assay showing the activity of the human *TP53* promoter construct (pGL2-200 bp) in CIAPIN1 overexpressing VSMCs pretreated with NAC (5 mM) for 30 min and then stimulated with H₂O₂ (100 μM, 1 h) or PDGF-BB (30 ng/mL, 3 h) (n = 6 per group).** (C)** Representative H&E staining of carotid arteries from a BI rat model, showing neointimal formation in scramble control (Ctr), rCIAPIN1 (rCIA1), BI + Ctr, BI + rCIA1 and BI + rCIA1 + NAC groups. Scale bar: 100 μm. I: intima, M: media. **(D)** Quantification of intima area (left) and intima-to-media ratio (right) in carotid arteries from the groups shown in (C). Data represent mean ± S.E.M. values of four independent experiments. **(E)** Representative immunofluorescence images of dihydroethidium (DHE, red), CIAPIN1 (green) and nuclei (DAPI, blue) in carotid arteries from BI + rCIA1 and BI + rCIA1 + NAC groups. **(F)** Quantification of DHE and CIAPIN1 expression from the groups shown in (E). Scale bar: 100 μm. ^**^p < 0.01,^***^p < 0.001, ^****^p < 0.0001 *vs*. each group. n.s.: not significant.

Based on our observation that PDGF-BB-induced ROS facilitates CIAPIN1 nuclear translocation and enhances its role in *Tp53* transcription suppression, we hypothesized that both increased CIAPIN1 expression and ROS generation are critical for the downregulation of *Tp53* transcription. To validate this, we evaluated *Tp53* transcription in VSMCs under various conditions. PDGF-BB treatment significantly reduced *Tp53* transcription, whereas H₂O₂ treatment alone had no discernible effect (Figure 4A), considering absence of CIAPIN1 expression (Figure 3C). Co-treatment with NAC completely abolished PDGF-BB's inhibitory effect on *Tp53* transcription in Ctr VSMCs (PDGF-BB+NAC), underscoring the role of ROS in this process. To further clarify the direct contribution of ROS, we overexpressed CIAPIN1 in VSMCs via hCIAPIN1.

**Figure 5:**
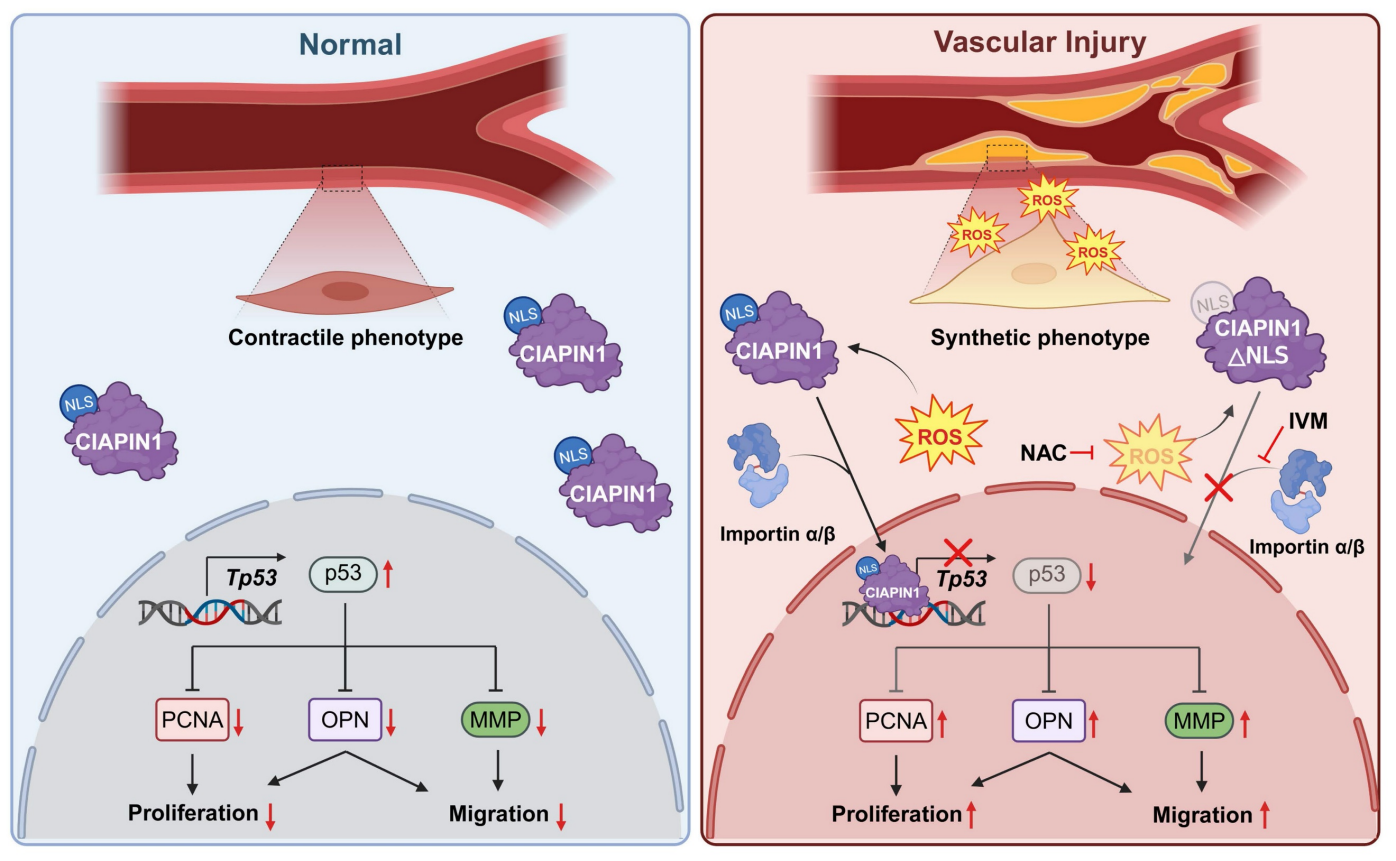
** Schematic of the mechanism by which pharmacological inhibition of CIAPIN1 nuclear translocation attenuates vascular hyperplasia**.

Under this condition, H₂O₂ or PDGF-BB alone markedly suppressed *Tp53* transcription unlike in H₂O₂-induced Ctr VSMCs (Figure 4A), and this effect was reversed by NAC co-treatment. We next performed a dual-luciferase assay to assess *Tp53* promoter activity in VSMCs that CIAPIN1 overexpression. Under basal conditions without PDGF-BB or H₂O₂ activation, *Tp53* transcription remained unchanged even though CIAPIN1 overexpression, but stimulation with either PDGF-BB or H₂O₂ markedly suppressed *Tp53* transcription, and this effect was abrogated by NAC treatment (Figure 4B). To further substantiate the functional consequence of CIAPIN1's nuclear translocation, we examined whether blocking its entry into the nucleus could reverse the suppression of p53 and the activation of VSMCs. Real-time PCR analysis demonstrated that the CIAPIN1-mediated downregulation of *Tp53* mRNA levels under PDGF-BB stimulation was significantly restored not only by NAC but also by pharmacological inhibition of the importin-α/β pathway using ivermectin or by genetic deletion of the identified NLS (∆236-239) (Figure S8A). Functional assays further corroborated these findings. Specifically, the marked increase in VSMC invasion observed in CIAPIN1-overexpressing cells was effectively neutralized when nuclear translocation was blocked via ivermectin treatment or NLS deletion (Figure S8B). Consistently, MTT assays revealed that the enhanced proliferation rate induced by CIAPIN1 overexpression was significantly attenuated when nuclear trafficking was inhibited by various approaches, including ROS scavenging, importin-α/β inhibition, or NLS mutation (Figure S8C). To investigate the *in vivo* relevance of these findings, we examined the role of CIAPIN1 and ROS in neointima formation using a rat model of BI. H&E staining of carotid artery sections revealed that BI significantly increased neointima formation compared to uninjured controls. Overexpression of CIAPIN1 in combination with BI, but not CIAPIN1 overexpression alone, further exacerbated neointima formation, suggesting that elevated CIAPIN1 levels synergize with vascular injury to promote vascular remodeling. However, treatment with NAC, a ROS scavenger, significantly attenuated BI-induced neointima formation in CIAPIN1-overexpressing aortas (Figure 4C-D). To further examine the role of ROS in this process, we performed dihydroethidium (DHE) staining to assess ROS levels in the arterial walls of post-BI. CIAPIN1 overexpression was associated with increased neointima formation and higher CIAPIN1 expression, consistent with its role in promoting VSMC dynamics. Treatment with NAC in CIAPIN1-overexpressing rats (rCIA1 + NAC) significantly reduced both ROS levels and neointima formation. Despite comparable levels of CIAPIN1 expression in both NAC-treated and untreated groups, NAC treatment inhibited the nuclear translocation of CIAPIN1, as indicated by reduced nuclear CIAPIN1 staining and a lower Pearson correlation coefficient (Figure 4E-F). Collectively, these results indicate that PDGF-BB-induced ROS generation promotes CIAPIN1 nuclear translocation and enhances *Tp53* transcription suppression, thereby exacerbating neointima formation *in vivo*. The similar protective effects observed with ROS scavenging, importin-α/β inhibition, and NLS deletion further establish nuclear translocation as a critical determinant of CIAPIN1 pathogenic function (Figure 5, Figure S8). Together, these findings suggest that disrupting ROS-dependent CIAPIN1 nuclear trafficking may represent a promising therapeutic strategy for vascular remodeling.

## Discussion

Vascular remodeling, driven by the phenotypic switching of vascular smooth muscle cells (VSMCs), is a central pathological process underlying atherosclerosis and restenosis 19, 38-40. While the pivotal role of p53 in maintaining VSMC homeostasis has been recognized for decades, the precise molecular circuits that suppress p53 expression under oxidative stress have remained elusive. In the present study, we identify a previously unrecognized PDGF-BB/ROS/CIAPIN1/p53 signaling axis that contributes to pathological vascular remodeling. Our data suggest that redox-sensitive nuclear translocation of CIAPIN1 contributes to VSMC phenotypic switching by directly repressing *Tp53* transcription.

Our findings extend previous observations regarding the role of CIAPIN1 in VSMCs by providing additional mechanistic insight 15. Although CIAPIN1 was recently identified as a modulator of VSMC activation, its subcellular localization and direct downstream targets had not been characterized 25, 41. While earlier studies have primarily described CIAPIN1 as a cytosolic protein 15, 17, we demonstrate here that it undergoes robust nuclear translocation in response to PDGF-BB-induced ROS generation. ChIP assays further confirmed this nuclear role, showing direct binding of CIAPIN1 to specific consensus sites in the *Tp53* promoter (Figure 4G). These results support a revised model in which CIAPIN1 function as a direct transcriptional regulator of cellular homeostasis, beyond its previously recognized role as a signaling adaptor.

We have also delineated the molecular machinery that drives CIAPIN1 nuclear trafficking downstream of PDGF-BB-induced oxidative signaling 42. Sequence analysis identified a classical nuclear localization signal (NLS; KKRK) at residues 236-239 in the C-terminal domain (Figure S7A) 43. Nuclear entry is mediated by the classical importin-α/β pathway 44, as pharmacological inhibition using ivermectin or genetic deletion of the NLS (Δ236-239) markedly attenuated ROS-driven nuclear accumulation of CIAPIN1 and restored *Tp53* expression (Figures S7-8). Although the anti-proliferative effects of the ROS scavenger NAC are well established in vascular injury models 45-47, our study provides a specific molecular explanation for this phenomenon. NAC-mediated ROS scavenging appears to prevent CIAPIN1 nuclear translocation, thereby maintaining p53-dependent homeostasis. These findings indicate that selectively targeting CIAPIN1 nuclear translocation—rather than broadly scavenging ROS—could represent a more precise and potentially therapeutic approach for proliferative vascular disorders.

Nevertheless, additional layers of complexity are likely involved in this process. ROS may promote CIAPIN1 nuclear trafficking through post-translational modifications, such as cysteine oxidation 48, that alter its conformation and interaction with the importin machinery. Identifying the redox-sensitive residues involved in this process will require further investigation. Moreover, although the present study establishes the CIAPIN1/p53 axis in VSMCs, potential compensatory pathways and additional transcriptional targets of CIAPIN1 in other vascular cell types remain to be explored.

In conclusion, integration of oxidative stress signals through CIAPIN1 nuclear translocation leads to direct transcriptional repression of *Tp53*, thereby promoting VSMC phenotypic switching and neointimal hyperplasia. These findings broaden our understanding of redox-sensitive transcriptional control in vascular plasticity and suggest that combined targeting of CIAPIN1 nuclear translocation and ROS signaling may provide a potential therapeutic strategy for proliferative vascular disorders.

## Supplementary Material

Supplementary figures and tables.

## Abbreviations

BI: balloon injury
CIAPIN1: cytokine induced apoptosis inhibitor 1
ChIP: chromatin immunoprecipitation
ECM: extracellular matrix
GSH: glutathione
IVM: ivermectin
JAK2/STAT3: Janus Kinase 2/Signal Transducer and Activator of Transcription 3
MMP-2: matrix metalloproteinase-2
NAC: N-acetylcysteine
NLS: nuclear localization signal
NOX1: NADPH oxidase 1
OPN: osteopontin
PCNA: proliferating cell nuclear antigen
