# Supplementary material for: CIAPIN1 functions as a redox-sensitive transcriptional repressor of Tp53 during vascular remodeling
Source: Theranostics. 2026 Apr 23;16(11):6350–65. doi: 10.7150/thno.124965 (PMC13142666; doi:10.7150/thno.124965)
Supplement: Supplementary file 1 — Supplementary figures and tables. [file thnov16p6350s1.pdf]

Supplementary Information

CIAPIN1 functions as a redox-sensitive transcriptional repressor of *Tp53* during vascular remodeling

Seongpyo Lee[1,2], Joo-Hui Han[1,2*]

[1]College of Pharmacy, Woosuk University, Wanju 55338, Republic of Korea

[2]College of Pharmacy, Chungbuk National University, Cheongju 28160, Republic of Korea

Correspondence

Joo-Hui Han, College of Pharmacy, Chungbuk National University, Cheongju 28160, Republic of Korea

E-mail: joohui.han@chungbuk.ac.kr

Table S1. Antibodies information

**Antibody**	**Products information**	**Working concentration**
CIAPIN1	Atlas antibodies, HPA042182	1:1,000 for western blots<br>1:250 for immunofluorescence *in vivo*<br>1:100 for immunofluorescence *in vitro*
PCNA	Abfrontier, LF-MA50083	1:1,000 for western blots
β-actin	Abfrontier, LF-PA0207	1:2,000 for western blots
MMP-2	Milipore, AB19015	1:1,000 for western blots
p53	Cell Signaling Technology, 2524	1:1,000 for western blots
p21	Calbiochem, OP79-100	1:500 for western blots
OPN	Santa Cruz Biotechnology, sc-73631	1:1,000 for western blots
LaminA/C	Cell Signaling Technology, 4777	1:1,000 for western blots
Goat Anti-Rabbit	Cell Signaling Technology, 7074	1:2,000 for western blots
Goat Anti-Mouse	Abfrontier, LF-SA8001	1:2,000 for western blots
Anti-rabbit-FITC	Sigma-Aldrich, F0382	1:200 for immunofluorescence *in vivo*<br>1:100 for immunofluorescence *in vitro*
Anti-mouse-TRITC	Sigma-Aldrich, T7782	1:200 for immunofluorescence *in vivo*<br>1:100 for immunofluorescence *in vitro*

Table S2. si-RNA information

**Genes**	**Sense (5`-3`)**	**Antisense (5`-3`)**
Rat *Tp53* #1	GUCAUGGAGGAUUCACAGU	ACUGUGAAUCCUCCAUGAC
Rat *Tp53* #2	GGUCACCUAAUUCCAUGGA	UCCAUGGAAUUAGGUGACC

Table S3. Primers for real-time PCR

**Genes**	**Sense primers (5`-3`)**	**Antisense primers (5`-3`)**
Human *ACTB*, Rat *Actb*	TCCATCATGAAGTGTGACGT	GCTCAGGAGGAGCAATGAT
Rat *Ciapin1*	GTAAGAACTGCACCTGTGGC	CCTGGAGGTTGCTACTGCTC

Human *CIAPIN1*	AGTTTGTGGCAGTGGTCTGG	TGGGACTAAACATTGCAACAGC
Rat *Tp53*	GTCTACGTCCCGCCATAAAA	AGGCAGTGAAGGGACTAGCA
Human *TP53*	CTGGATTGGCAGCCAGACT	TCCGGGGACAGCATCAAATC

Table S4. ChIP-PCR primers for *Tp53* binding sites

Genes	Sense primers (5`-3`)	Antisense primers (5`-3`)
-330 to -31	GCCGCACTTAAAATAGATCGTAAAAGC	TGGGAGGGGAAAGTCCCAATCC
-330 to -181	CTCAAGCAGAACCCTGACTCTGCA	ATGTTGCCGCCAGCACGAACGCTT
-181 to -31	CTCAAGCAGAACCCTGACTCTGCA	TGGGAGGGGAAAGTCCCAATCC

Table S5. Primers for mutagenesis

**Mutation**	**Mutagenesis primer (5’ to 3’)**
CIAPIN1	F: 5’-CCC AGA ATT CAT TAA AGA GGA GAA ATT AAC TAT GAA ACA TC-3<br>R: 5’-GGC CGG CCG CTC AGG C-3
△S236-239	F: 5’-GGC CTG TAA GAA CCT TGC CGA AGA AC-3’<br>R: 5’-GTT CTT CGG CAA GGT TCT TAC AGG CC-3’

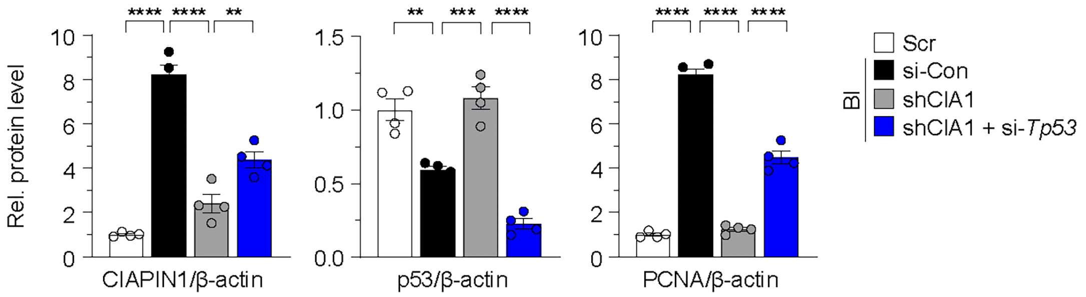

Figure S1. CIAPIN1 knockdown reverses balloon injury-induced changes in CIAPIN1, p53, and PCNA expression in arteries. Quantitative analysis of protein levels in Figure 1C (n = 4 per group). $^{**}p < 0.01$, $^{***}p < 0.001$ and $^{****}p < 0.0001$ *vs* each group. Data represent mean ± S.E.M. values of four independent experiments.

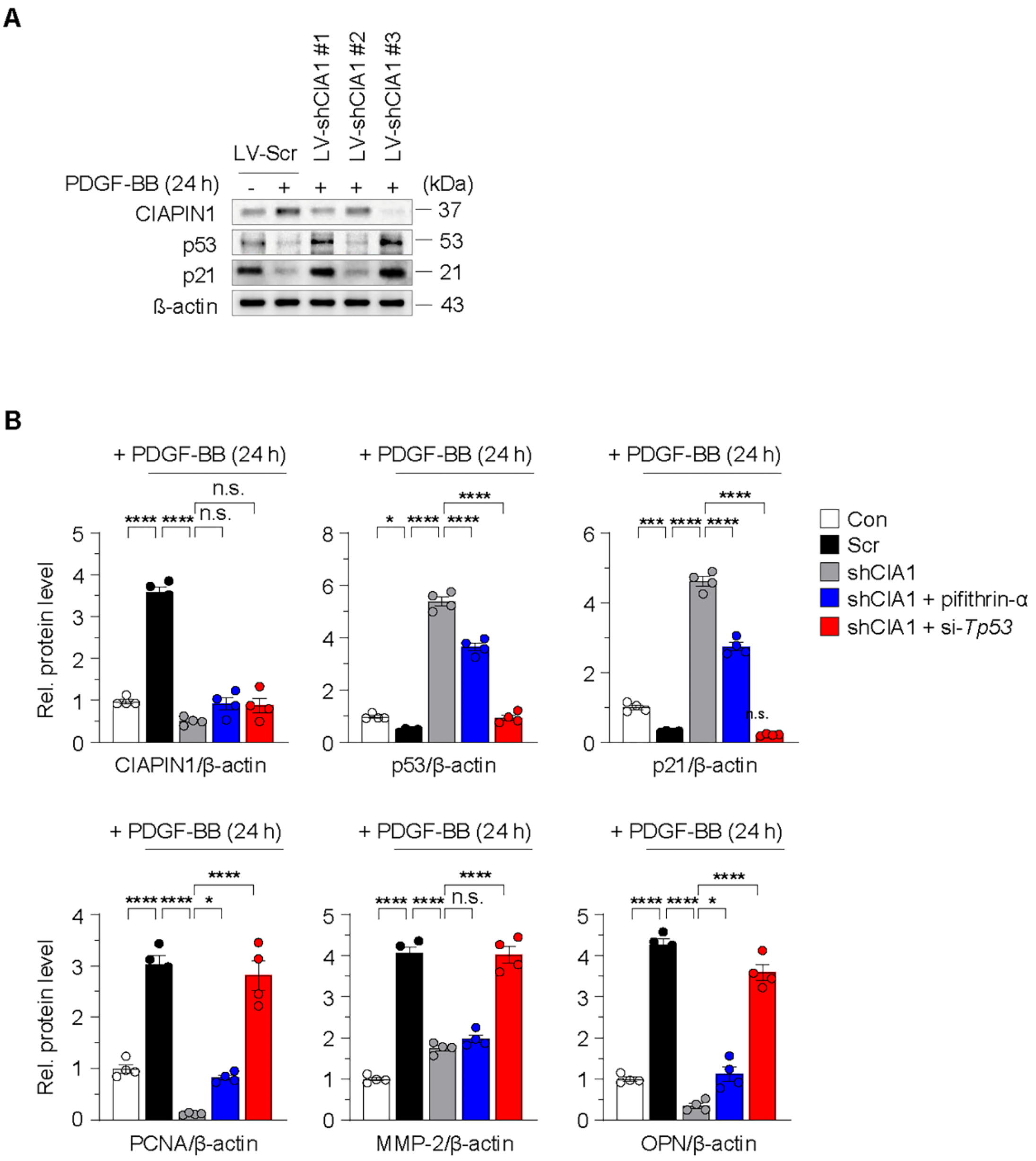

Figure S2. CIAPIN1 knockdown reverses PDGF-BB-induced changes in p53, and p21 expression in VSMCs. (A) Western blot analysis of CIAPIN1, p53 and p21 protein levels in VSMCs. Cells were transduced with lentiviral vectors expressing scramble control (LV-Scr) or CIAPIN1 shRNA (LV-shCIAPIN1 #1, #2, #3) and treated with or without PDGF-BB (30 ng/mL) for 24 h. β-actin was used as a loading control. **(B)** Quantitative analysis of protein levels in Figure 1G (n = 4 per group). $^{**}p < 0.01$, $^{***}p < 0.001$ and $^{****}p < 0.0001$ *vs* each group. n.s.: not significant. Data represent mean ± S.E.M. values of four independent experiments.

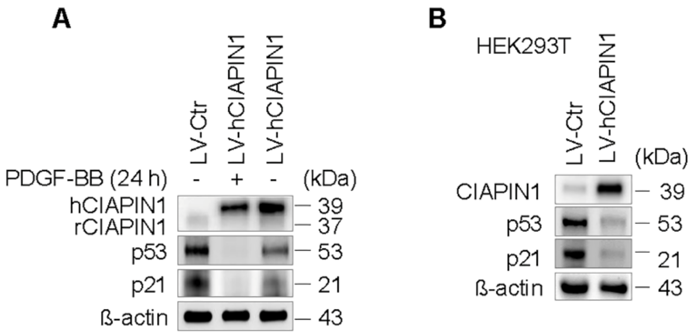

Figure S3. CIAPIN1 overexpression enhances suppression of p53 and p21 in VSMCs and HASMCs. (A) Western blot analysis of CIAPIN1, p53 and p21 protein levels in CIAPIN1 overexpressing VSMCs after stimulation with PDGF-BB (30 ng/mL) for 24 h. **(B)** Western blot analysis of CIAPIN1, p53 and p21 protein levels in CIAPIN1 overexpressing HEK293T cells. β-actin was used as a loading control.

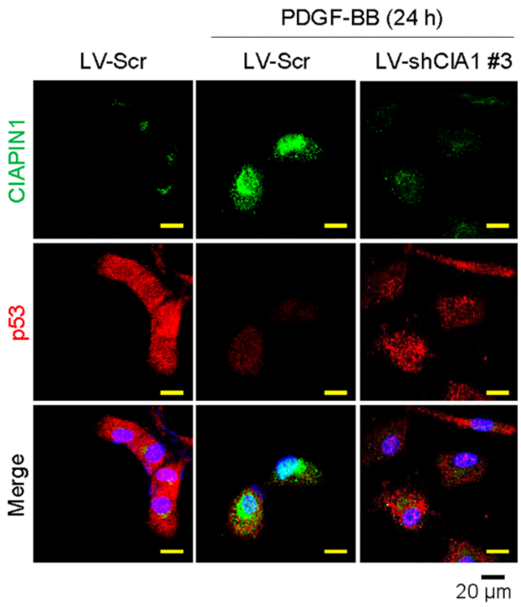

Figure S4. CIAPIN1 knockdown recovers the p53 expression reduced by PDGF-BB. Representative immunofluorescence images of CIAPIN1 (green) and p53 (red) in CIAPIN1 knockdown VSMCs treated with PDGF-BB (30 ng/mL) for 24 h. Nuclei were stained with DAPI (blue). Scale bar: 20 μm.

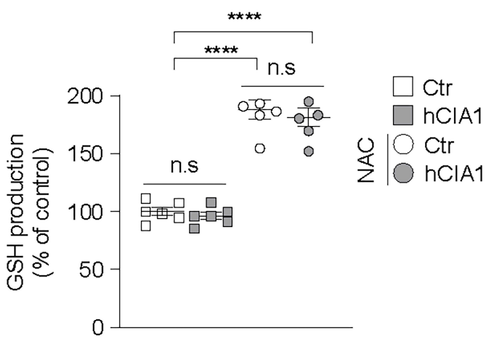

Figure S5. CIAPIN1 overexpression has no effect on GSH levels. GSH levels in CIAPIN1 overexpressing VSMCs treated with NAC (5 mM) for 30 min (n = 6 per group). $^{****}p < 0.0001$ *vs*. each group. n.s.: not significant.

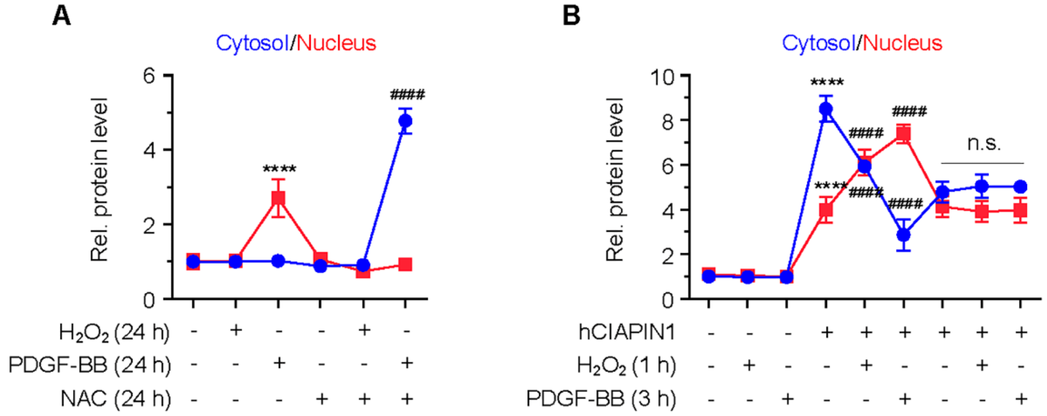

Figure S6. ROS facilitates the CIAPIN1 translocation from cytosol into nucleus. (A) Quantitative analysis of protein levels in Figure 3C (n = 4 per group). $^{****}p < 0.0001$ *vs* Con, $^{####}p < 0.0001$ *vs* NAC. **(B)** Quantitative analysis of protein levels in Figure 3D (n = 4 per group). $^{****}p < 0.0001$ *vs* Ctr, $^{####}p < 0.0001$ *vs* hCIAPIN1. n.s.: not significant. Data represent mean ± S.E.M. values of four independent experiments.

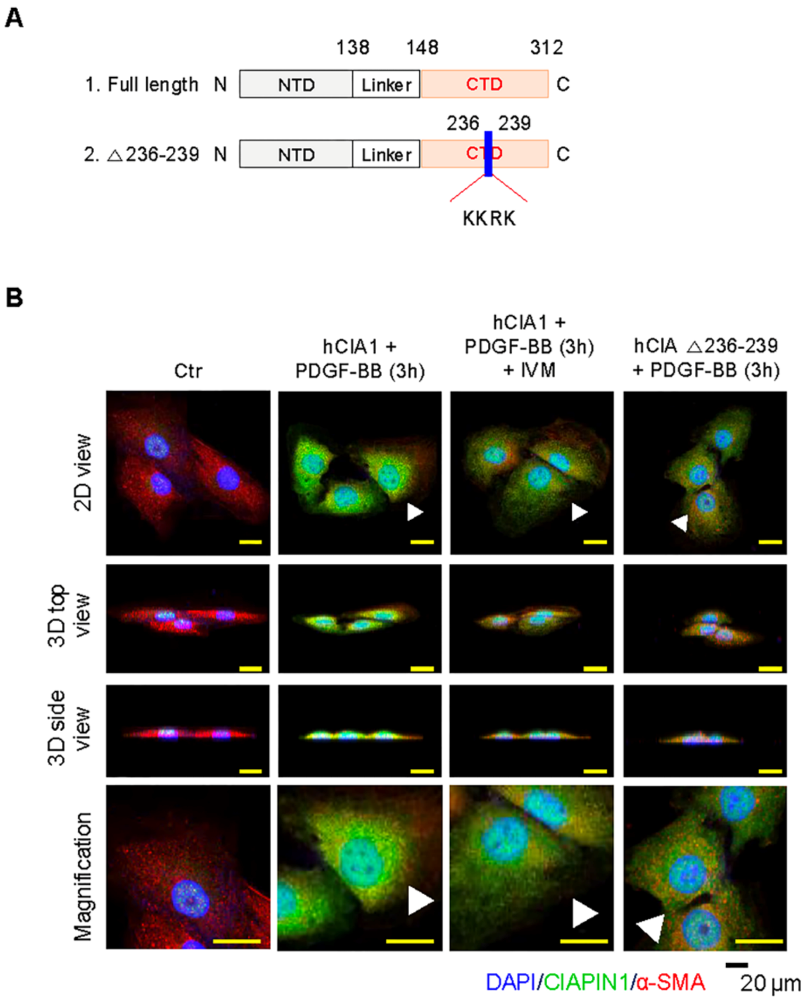

Figure S7. CIAPIN1 undergoes ROS-dependent nuclear translocation via NLS through classical importin- α/β pathway. (A) Schematic illustration of the domain structure of full-length human CIAPIN1 (aa 1–312) and the NLS-deletion mutant (Δ236-KKRK-239; K: Lysine; R: Arginine). **(B)** Representative Z-stack immunofluorescence images showing the localization of CIAPIN1 (green) and α-SMA (red) in VSMCs. To investigate the mechanism of CIAPIN1 nuclear translocation, cells were pretreated with ivermectin (IVM, 25 μM), an inhibitor of the importin-α/β pathway, for 30 min prior to stimulation with PDGF-BB (30 ng/mL) for 3 h. In addition, VSMCs were transfected with the NLS-deleted CIAPIN1 mutant (Δ236–239) to determine the requirement of this region for nuclear translocation. Nuclei were counterstained

with DAPI (blue). Images are presented as 2D views, 3D top views, 3D side views, and magnified views. Scale bars: 20 μm.

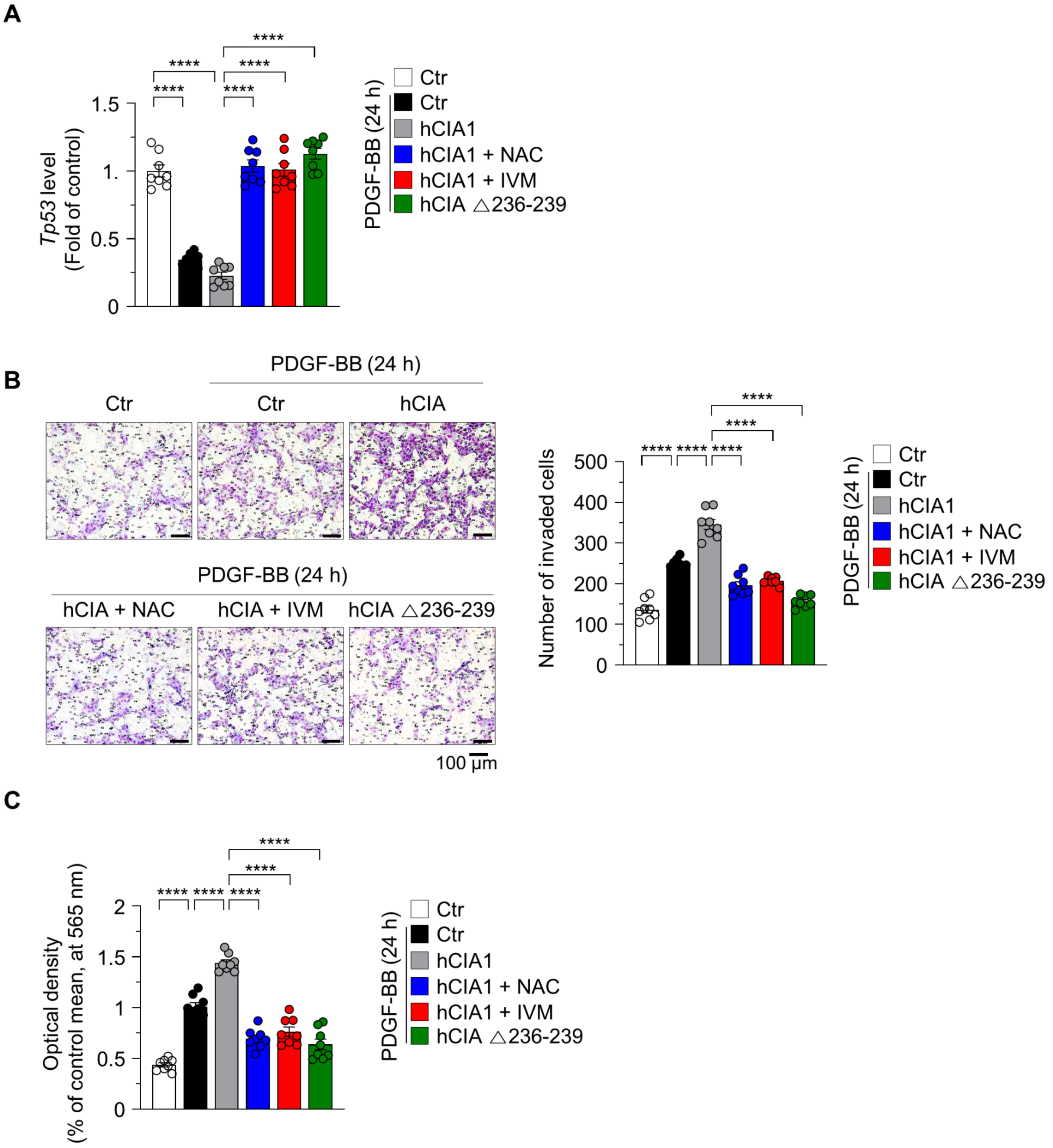

Figure S8. Blockade of CIAPIN1 nuclear entry by ROS scavenging, NLS deletion, or inhibition of the importin-α/β pathway suppresses p53-dependent vascular remodeling. Effects on **(A)** *Tp53* mRNA expression, **(B)** cell migration, and **(C)** cell proliferation were examined in VSMCs treated with NAC, ivermectin (IVM), or the NLS-deleted CIAPIN1 mutant (Δ236–239). Cells were stimulated with PDGF-BB (30 ng/mL) for 24 h after pretreatment with NAC (5 mM) or IVM (25 μM), or after transfection with the NLS-deleted CIAPIN1 mutant (Δ236–239) (n = 8 per group). *Tp53* mRNA levels, cell migration, and

proliferation were assessed by real-time PCR, transwell migration assay, and MTT assay, respectively. Scale bar, 100 μm.
